# Comparison of refractive outcomes between femtosecond laser-assisted cataract surgery and conventional cataract surgery

**DOI:** 10.1097/MD.0000000000013784

**Published:** 2018-12-28

**Authors:** Woong-Joo Whang, Young-Sik Yoo, Choun-Ki Joo, Geunyoung Yoon

**Affiliations:** a Department of Ophthalmology and Visual Science, Yeouido St. Mary's Hospital; b Department of Convergence Medical Science; c Department of Ophthalmology and Visual Science, Seoul St. Mary's Hospital, College of Medicine, The Catholic University of Korea, Seoul, South Korea; d Flaum Eye Institute, Center for Visual Science, The Institute of Optics, University of Rochester, Rochester, New York, USA.

**Keywords:** femtoemulsification, IOL formula, predictive accuracy, refractive outcome

## Abstract

The purpose of our study is to compare the predictive accuracy of femtosecond laser-assisted cataract surgery with the results of conventional cataract surgery. This prospective study included 83 eyes from 83 patients who underwent femtosecond laser-assisted cataract surgery and 83 eyes from 83 patients who underwent conventional cataract surgery. Preoperative IOL power calculations were performed with the partical coherence interferometry. Femtosecond laser-assisted capsulotomy was based on 5.2 mm scanned capsule center. Following phacoemulsification, 1-piece IOL was inserted into the capsular bag. Refractive outcome was measured 3 months postoperatively with manual refraction. Predicted refraction was assessed by the Barret-Universal II, Haigis, Hoffer Q, SRK/T, and T2 formulas. We applied optimized IOL constants and retrospectively personalized IOL constants. There was no difference in preoperative demographic data. When the optimized IOL constants were used, the femtosecond laser-assisted group produced significantly lower MAEs in the Barret-Universal II, Hoffer Q, SRK/T, and T2 formulas (*P* < .05). After the personalization of IOL constants, there were statistical differences in the Barret-Universal II, Hoffer Q (*P* < .05). The standard deviation of ME and MedAE were also relatively lower with femtosecond laser-assisted group. In conclusion, femtosecond laser-assisted cataract surgery with Catalys femtosecond laser system produced better refractive outcomes than conventional cataract surgery.

## 1 Introduction

Femtosecond laser-assisted cataract surgery is a new technology with potential benefits and barriers that surgeons should be aware of when considering whether or not to integrate laser technology into their practice.^[1]^ Femtosecond laser produces a more predictable capsulotomy than conventional cataract surgery. The evidence suggests that femtosecond laser improves the capsulotomy shape, size, and centration, and it makes for better intraocular lens (IOL)/anterior capsule overlap.^[2–5]^

Lawless et al^[6]^ demonstrated that femtosecond laser-assisted cataract surgery does not improve predictability compared to conventional cataract surgery. However, Filkorn et al^[7]^ concluded that the femtosecond laser group showed a smaller mean absolute error (MAE) than conventional surgery. Roberts et al^[8]^ concluded that the mean absolute error in diopter was 0.29 ± 0.25 D for femtosecond laser-assisted cataract surgery and 0.31 ± 0.24 D for the manual group. While Abell et al^[9]^ found that femtosecond laser-assisted cataract surgery produced more myopic results than the manual cataract surgery (−0.51 ± 0.50 D versus −0.45 ± 0.71 D), the results may have been influenced by non-optimized IOL constants. The results of past studies are therefore contradictory and they had some limitations regarding an accurate comparison. Furthermore, no studies have investigated the predictive accuracy in the case of using the Catalys Precision laser system.

In the present study, we compared the refractive outcomes derived from femtosecond laser-assisted cataract surgery and conventional cataract surgery, as calculated by 5 kinds of IOL formulas (the Barret-Unversal II, Haigis, Hoffer Q, SRK/T, and T2 formulas).

## 2 Methods

This prospective study included 83 eyes from 83 patients who underwent femtosecond laser-assisted cataract surgery and 83 eyes from 83 patients who underwent conventional cataract surgery from Jan 2016 to Dec 2016. The exclusion criteria were previous ocular surgery, corneal diseases, pseudoexfoliation, zonular weakness, corneal astigmatism greater than 3.00 diopters, glaucoma, macular disease, and amblyopia. Eyes with best-corrected distant vision less than 20/40 at postoperative state were also excluded.

Informed consent was obtained from all of the patients before the commencement of the study, and the study methods adhered to the tenets of the Declaration of Helsinki for the use of human participants in biomedical research. The Institutional Review Board for Human studies, Seoul St. Mary hospital approved this study. (KC13DISI0534)

Preoperative IOL power calculations were performed with the IOLMaster optical biometer (version 5, Carl-Zeiss Meditec, Germany). The IOLMaster uses partial coherence interferometry to measure the axial length (AL). Corneal power is measured by automated keratometry, which should be performed 1st because the system requires the input of corneal radii to calculate the anterior chamber depth (ACD). The ACD is determined by calculating the distance along the visual axis between the corneal epithelium and the anterior lens surface using lateral slit illumination. Nemeth et al^[10]^ reported good reproducibility of the ACD measurements using the IOLMaster.

Three ophthalmologists participated in this study. The 1st ophthalmologist (W.J.W) enrolled patients and performed preoperative examination. The 2nd ophthalmologist (Y.S.Y) who was blinded to the patients’ information classified the patients into 2 groups. The 3rd ophthalmologist (C.K.J) performed the cataract surgery. After sufficient pupillary dilation was confirmed, femtosecond laser pretreatments were performed with the Catalys Precision Laser System (Abbott Medical Optics, Abbott Laboratories Inc., Abbott Park, IL) in patients in the femtosecond laser-assisted group. All of the laser treatment procedures were performed under topical anesthesia induced with 0.5% proparacaine hydrochloride (Alcaine, Alcon Laboratories, Fort Worth, TX). Before laser treatment, the capsulotomy diameter and centration method were chosen. The pattern of lens fragmentation and grid spacing were selected on the control panel of the laser system. The axis, width, length of the primary incision and side-port incision were also selected. The parameters of femtosecond laser pretreatment are described in Table 1.

**Table 1:**
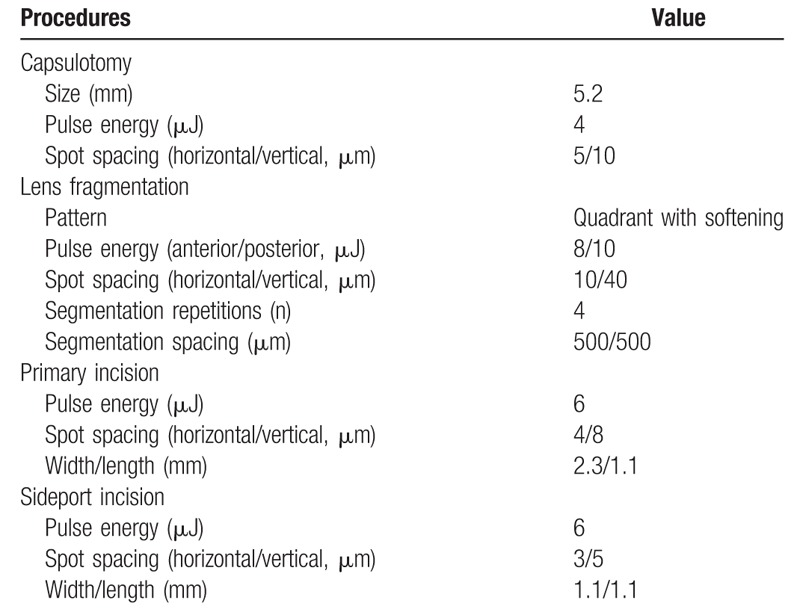
Parameters of femtosecond laser pretreatment.

The disposable vacuum interface (LIQUID OPTICS, Abbott Medical Optics, Abbott Laboratories Inc., Abbott Park, IL) was positioned and fixed to the globe using a suction ring and the laser aperture was engaged with the vacuum interface-globe complex. After completion of the entire laser emission procedure, the vacuum interface was removed and the patient was transported to a day-surgery operation room. At the initiation of cataract surgery, laser corneal incision sites (2.3 mm in width) at temporal side were carefully dissected with a Sinskey hook and the incised circular capsule (5.2 mm in diameter, scanned capsule type) was removed using micro-forceps. The patients in the conventional group underwent a 2.2 mm clear corneal temporal incision followed by continuous curvilinear capsulorhexis (CCC). We used Akaoshi CCC 5.5 marker (ASICO, IL) centered on dilated pupil. There was no complication during capsulotomy in both groups. Local anesthesia was administered using topical 4% lidocaine and 0.5% proparacaine hydrochloride (Alcaine, Alcon Laboratories, Fort Worth, TX) before phacoemulsification. All of the surgeries were performed using the Ozil torsional hand piece with the Infiniti Vision System (Alcon). Phacoemulsification was performed with 100% torsional ultrasound, 350 mm Hg vacuum, and a 35 cc/min aspiration rate. Following phacoemulsification, 1 type of intraocular lens (ZCB00, Johnson & Johnson Vision Care, Inc.) was inserted into the capsular bag in both groups. No intraoperative complications occurred.

Refractive outcome was measured 3 months postoperatively with manual refraction. Predicted refraction was assessed by the Barret-Universal II, Haigis, Hoffer Q, SRK/T, and T2 formulas. We applied optimized IOL constants published on the User Group for Laser Interference Biometry (ULIB) website (http://www.ocusoft.de/ulib/c1.htm). The mean values of intended target refraction for the Barret-Universal II, Haigis, Hoffer Q, SRK/T, and T2 formulas were −0.79 ± 0.95, −0.70 ± 0.97, −0.75 ± 0.99, −0.66 ± 0.92, −0.69 ± 0.91 diopter in femotosecond laser-assisted group and −0.77 ± 1.04, −0.71 ± 1.06, −0.74 ± 1.09, −0.59 ± 0.94, −0.65 ± 1.00 diopter in conventional group.

The refractive outcomes by 4 formulas (The Barret-Universal II, hoffer Q, SRK/T, and T2 formulas) were personalized retrospectively by adjusting each formula's IOL constant to give a mean error of zero in each group. The IOL constants of a0, a1, and a2 for the Haigis formula were calculated with linear regression analysis using the retrospectively calculated effective lens position (ELP) and the following thin-lens formula.
 
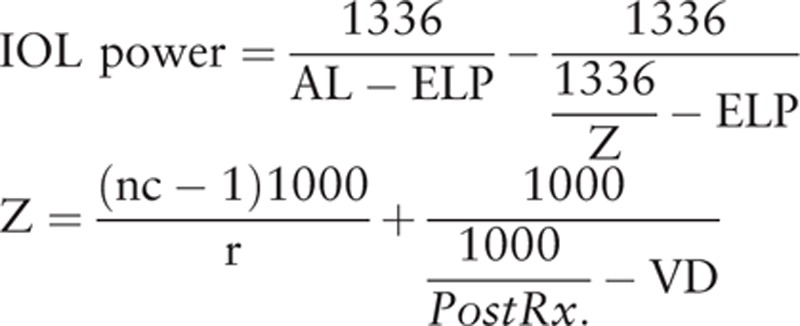


where AL is the axial length, ELP is the effective lens position, nc is the fictious corneal refractive index, r is the corneal radius, PostRx. is the postoperative refraction, and VD is the vertex distance.

Personalization was performed using Microsoft Excel program. The optimized IOL constants and personalized IOL constants were listed in Table 2. The mean error (ME) was the actual postoperative spherical equivalent (SE) minus the predicted SE. The mean absolute error (MAE) and median absolute error (MedAE) were the mean value and median value in the absolute value of the ME. We also calculated the percentage of eyes with a ME of  ± 0.25,  ± 0.50, and  ± 1.00 diopters or less. IOL tilt was also analyzed according to the method described by de Castro et al^[11]^ and Kranitz et al^[12]^ with a rotating Scheimpflug camera, the Pentacam HR (software version 1.17r91; Oculus; Wetzlar, Germany). This was used to analyze the cornea via a 25-picture scan; only scans graded as being “OK” by the instrument specifications were included in this study.

**Table 2:**
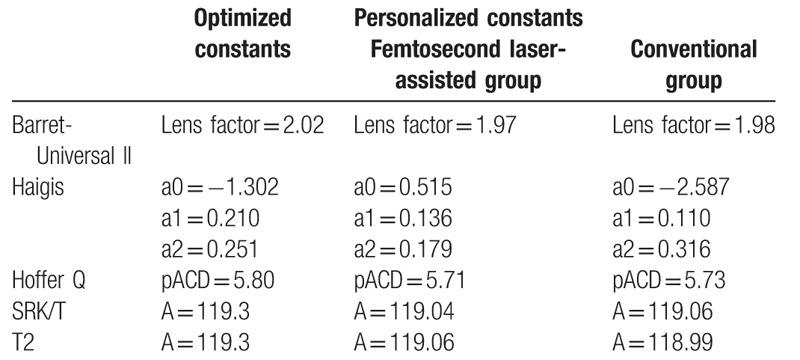
Optimized IOL constants and personalized IOL constants for the IOLMaster biometry.

A positive IOL tilt around the x-axis indicates that the superior edge of the IOL is located on the front, while a positive tilt around the y-axis indicates that the temporal edge of the IOL has moved backwards when compared with the nasal edge. We also calculated the mean absolute values of IOL tilt and compared the results between 2 groups.

Statistical analysis was performed using SPSS statistical software (version 19.0, SPSS, Inc). We determined the significance of the predictive accuracy and IOL tilt between the two groups, with the Mann–Whitney *U* test; we also performed the Chi-square test to compare the percentages of eyes with a ME of  ± 0.25,  ± 0.50, and  ± 1.00 diopters. The experimental level of significance was set at 0.05.

## 3 Results

We evaluated 166 eyes; patient characteristics and biometric data are shown in Table 3. Axial lengths in the femtosecond laser assisted group ranged from 21.41 to 28.55 mm (mean: 23.92 mm); in the conventional group, from 21.46 to 28.22 mm (mean: 23.90 mm). Preoperative ACD in the femtosecond laser assisted group ranged from 2.23 to 3.93 mm (mean: 3.15 mm); in the conventional group, from 2.32 to 4.02 mm (mean: 3.13 mm). Preoperative mean corneal powers in the femtosecond laser assisted group ranged from 41.00 to 48.03 D (mean: 44.02 D); in the conventional group, from 41.38 to 47.38 D (mean: 44.37 D). The refractive powers of implanted IOLs in the femtosecond laser assisted group ranged from 12.0 to 26.0 D (mean: 20.83 D); in the conventional group, from 11.0 to 26.0 D (mean: 20.43 D). There was no statistically significant difference in the demographic data between the 2 groups. Postoperative spherical equivalent also showed no difference between 2 groups. (−0.86 ± 1.00 D for femtosecond laser assisted group versus −0.83 ± 1.15 D for conventional group) While, both the horizontal tilt of IOL and the vertical tilt of IOL revealed the significant differences between 2 groups. (*P* < .001 for horizontal tilt; *P* = .004 for vertical tilt)

**Table 3:**
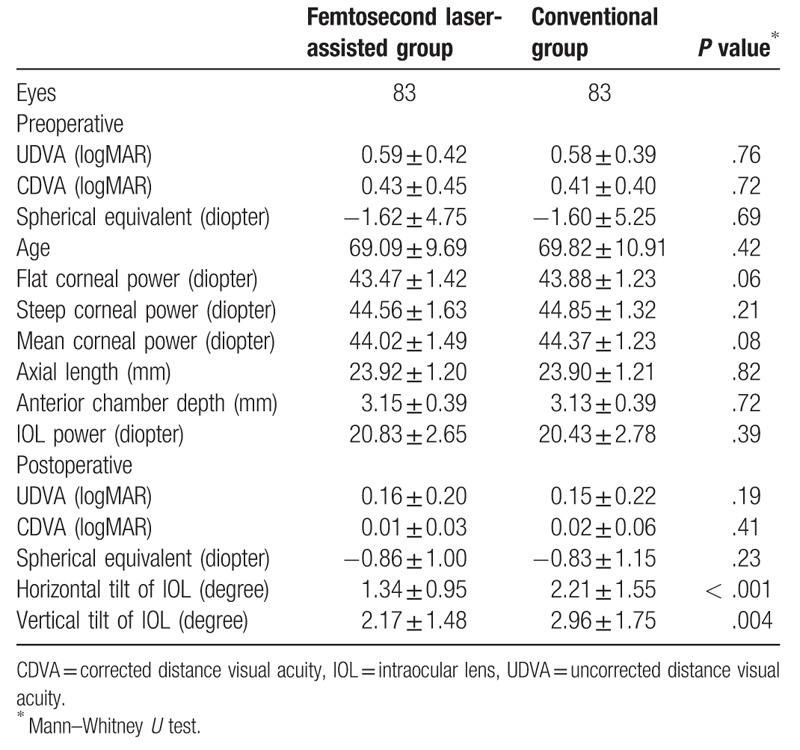
Patient characteristics and biometric data (axial length, anterior chamber depth, and corneal power) in the IOLMaster.

Table 4 shows refractive errors calculated by 5 formulas when the optimized IOL constants were applied. The absolute values of ME were not more than 0.25 D (−0.23 to −0.06) in both groups. The standard deviations of ME were 0.33 to 0.36 D in the femtosecond laser-assisted group; these values were less than the standard deviations of ME in the conventional group (0.45–0.51 D). The MAEs ranged from 0.28 to 0.32 D in the femtosecond laser-assisted group and from 0.37 to 0.45 D in the conventional group. The differences of MAEs in the Barret-Universal II, Hoffer Q, SRK/T, and T2 formulas were statistically significant (*P* < .05). The femtosecond laser-assisted group also yielded lower MedAEs than the conventional group in all formulas and the differences ranged from 0.02 to 0.12 D.

**Table 4:**
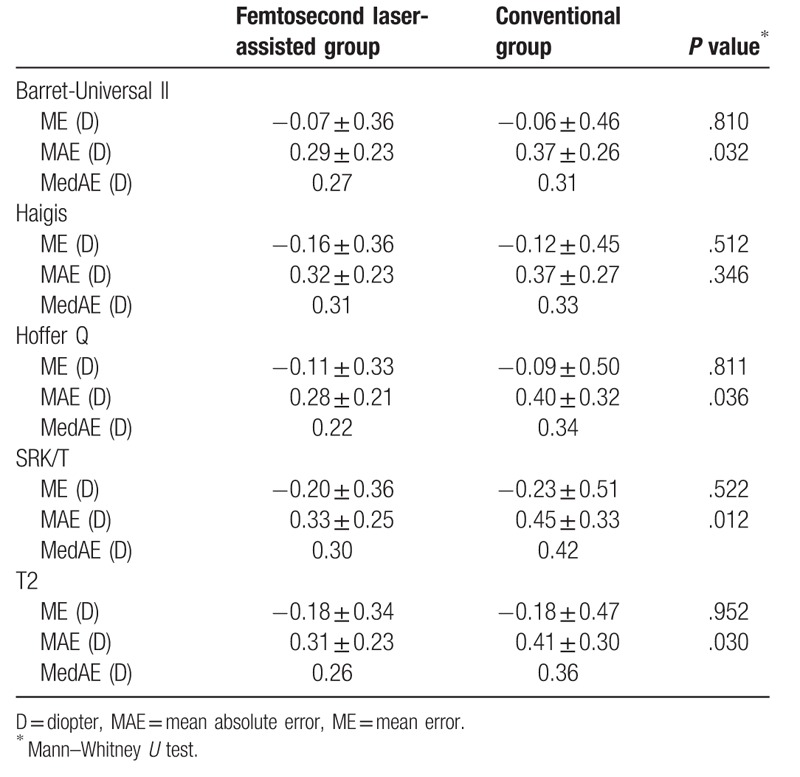
Mean (arithmetic) error and mean absolute errors in 2 groups when the optimized IOL constants were applied.

Table 5 shows the percentage of eyes with an error of prediction within ±0.25, ±0.50 and ±1.00 D when the optimized IOL constants were used. These values were higher in the femtosecond laser-assisted group than in the conventional group (within 0.25 D, 42.2–54.2% vs 37.3–41.0%; within 0.5 D, 77.1–81.9% vs 62.7–73.5%; within 1.0 D, 97.6–100% vs 91.6–96.4%). Especially, the femtosecond laser-assisted group yielded significantly higher percentages within 0.50 D in the Hoffer Q and SRK/T formula and within 1.00 D in the Hoffer Q formula.

**Table 5:**
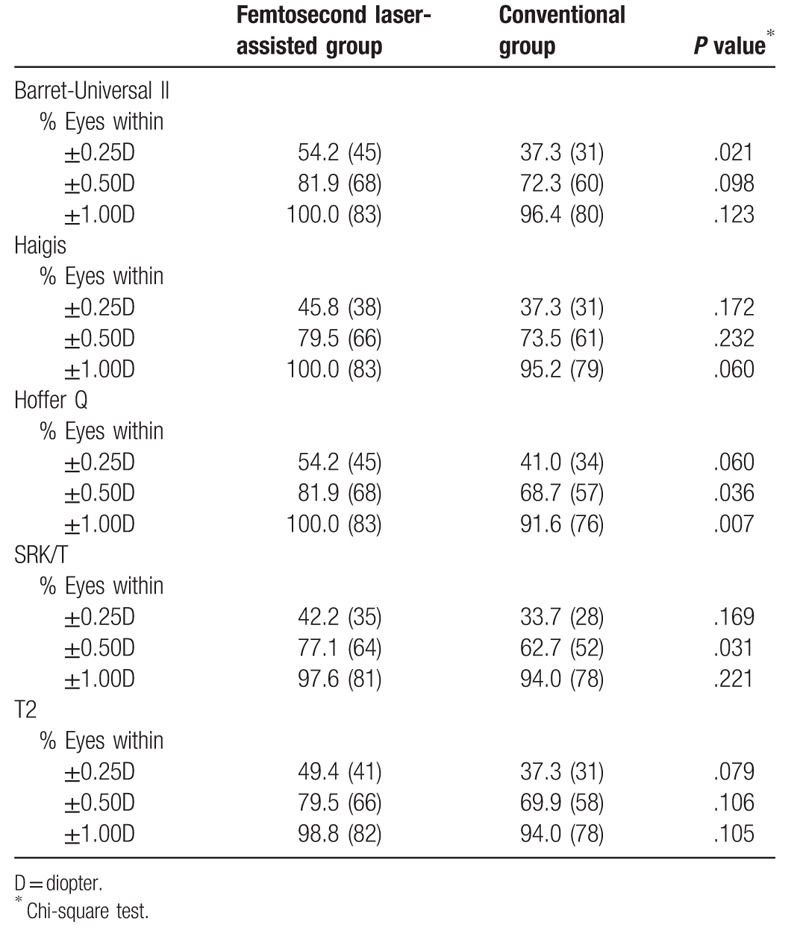
Percentage of eyes with an error of prediction of ±0.25, ±0.50, and ±1.00 diopter when the optimized IOL constants were applied.

Table 6 shows the results when the personalized IOL constants were used. The MAEs ranged from 0.26 to 0.29 D in the femtosecond laser-assisted group and from 0.35 to 0.40 D in the conventional group. The femtosecond laser-assisted group produced significantly lower MAEs in the Barret-Universal II and Hoffer Q formulas. (*P* < .05). The MedAEs were also lower in the femtosecond laser-assisted group and the differences ranged from 0.05 to 0.11 D.

**Table 6:**
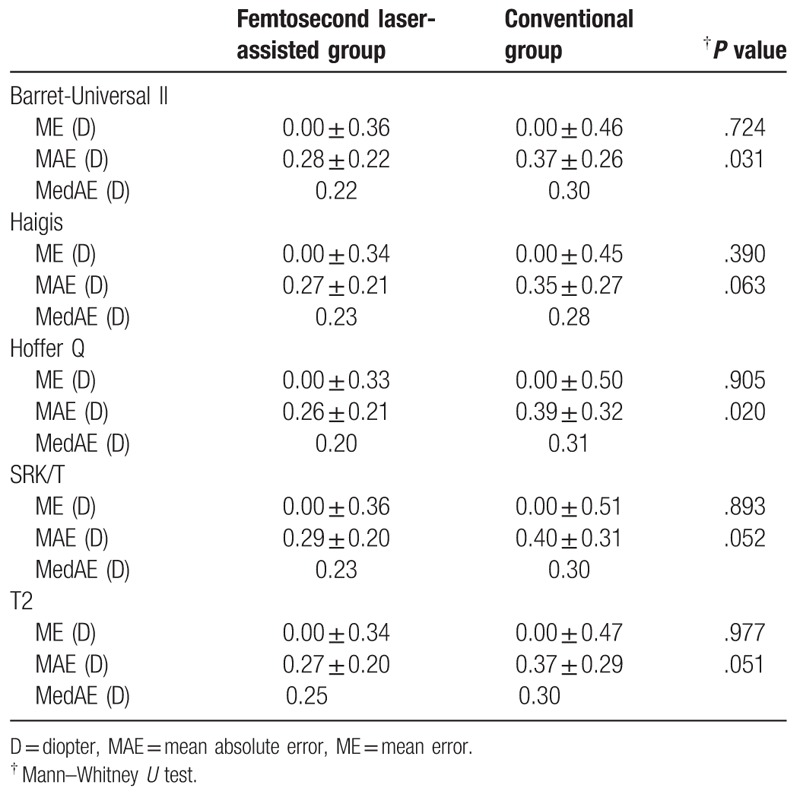
Mean (arithmetic) error and mean absolute errors in 2 groups when the retrospectively personalized IOL constants were applied.

The percentage of eyes with an error of prediction within ±0.25, ±0.50 and ±1.00 D were listed in Table 7. The femtosecond laser-assisted group showed higher percentages within ±0.25, ±0.50 and ±1.00 D. (within 0.25 D, 49.4–55.4% vs 37.3–45.8%; within 0.5 D, 83.1–89.2% vs 69.9–73.5%; within 1.0 D, 100% vs 92.8–98.8%). There was no case where the refractive error was more than 1.00 D in the femtosecond laser-assisted group and the percentages within 0.50 D were statistically higher with femtosecond laser-assisted group except when using the Barett-universal II formula.

**Table 7:**
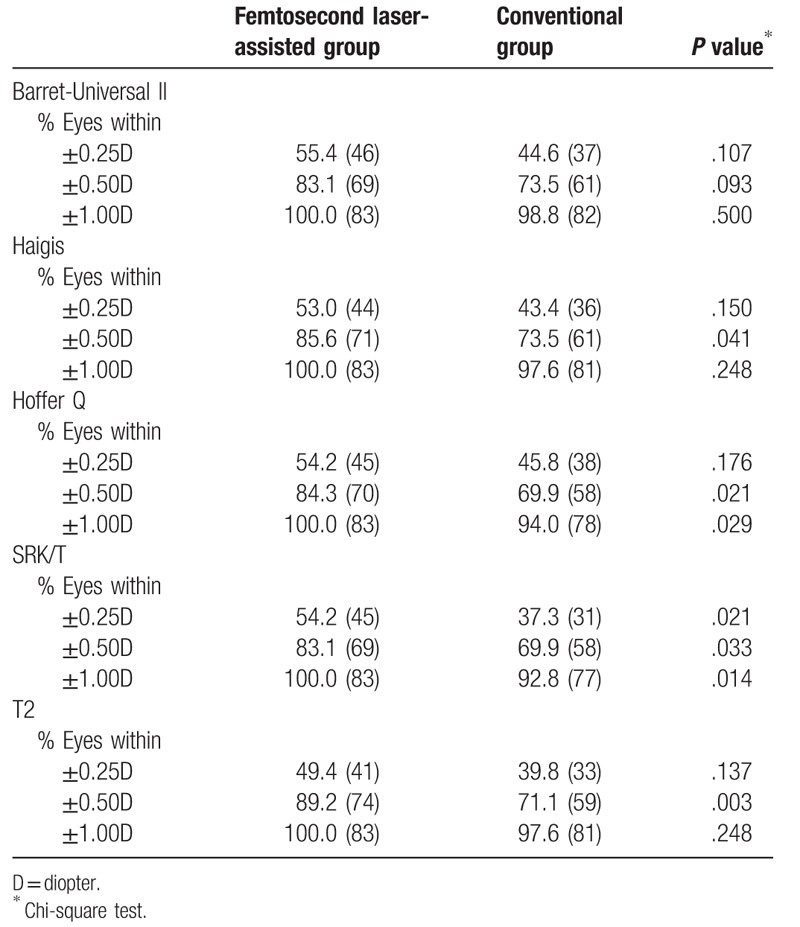
Percentage of eyes with an error of prediction of ±0.25, ±0.50, and ±1.00 diopter when the retrospectively personalized IOL constants were applied.

### 3.1 Discussion

Our results show that femtosecond laser-assisted cataract surgery produces better refractive outcomes than conventional cataract surgery. The femtosecond laser-assisted group were produced significantly lower MAE than the conventional group. The percentage of eyes with an error of prediction within ±0.50 diopter was also statistically greater in femtosecond laser-assisted group. Furthermore, both the MedAE and the standard deviation of ME and were also lower.

**Figure 1:**
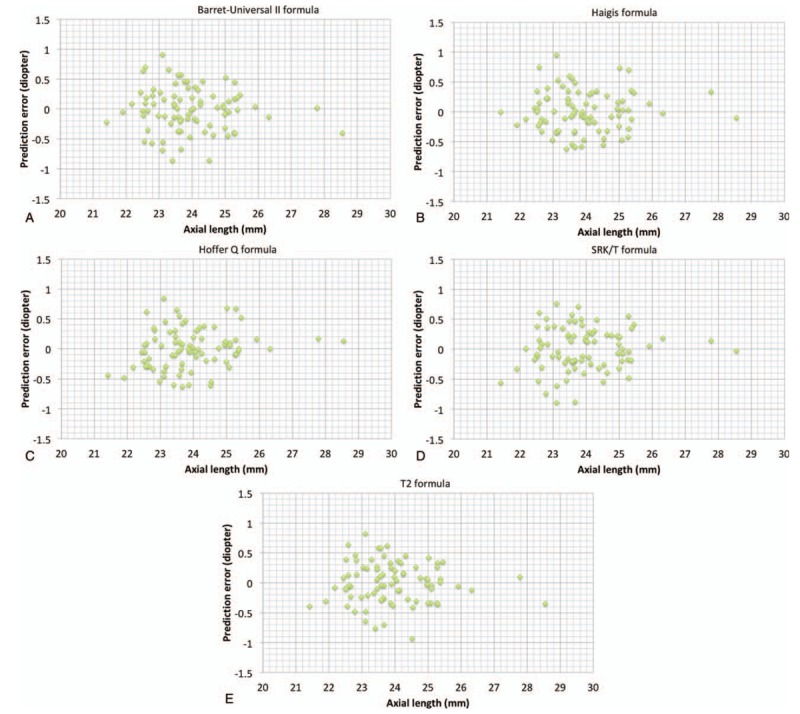
Preoperative axial length and predictive accuracy by 5 formulas in femtosecond laser-assisted group. (**A**)Barret-Universal II formula; (**B**)Haigis formula; (**C**) Hoffer Q formula; (**D**) SRK/T formula; (**E**)T2 formula.

**Figure 2:**
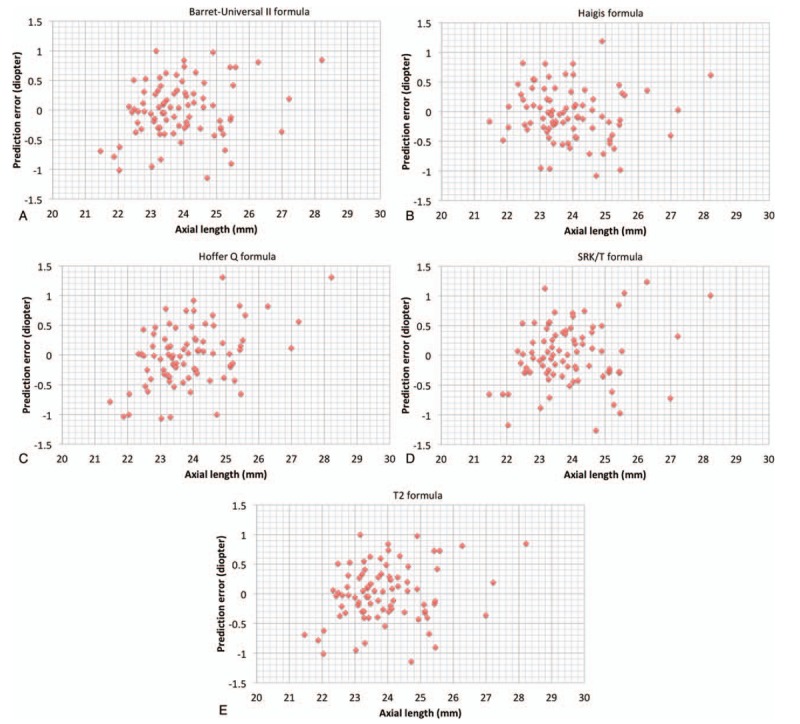
Preoperative axial length and predictive accuracy by 5 formulas in conventional group. (**A**)Barret-Universal II formula; (**B**)Haigis formula; (**C**) Hoffer Q formula; (**D**) SRK/T formula; (**E**)T2 formula.

Variations in capsulotomy size, shape, and position induce intraocular lens tilt, decentration, and anteroposterior movement and they contribute to variations in effective lens position.^[7]^ The error in IOL power calculation is most likely due to the variability of the effective lens position after cataract surgery.^[13]^ Manual capsulorhexis is considered to be the most difficult step in cataract surgery because it needs individual skills and experience. Femtosecond laser-created capsulotomy will produce smaller variations to enhance the stability of refractive outcomes. In a previous study using a Scheimpflug rotating camera, the risk of IOL decentration was 6 times higher with manual capsulorhexis than with femtosecond laser-created capsulotomy.^[12]^ In this study, we also analyzed IOL tilt in 2 groups and femtosecond laser-assisted group showed significantly smaller IOL tilt. The absolute value of the prediction error exceeded 1 diopter in at least 1 of the 5 formulas in 10 out of 83 eyes with manual CCC. (The Barret-Universal II: 1 eye; Haigis: 2 eyes; Hoffer Q: 5 eyes; SRK/T: 6 eyes; T2: 2 eyes) 10 eyes showed greater degrees in both horizontal tilt and vertical tilt (3.70 ± 1.70 vs 2.00 ± 1.41 for horizontal tilt; 4.10 ± 1.85 vs 2.81 ± 1.67 for vertical tilt) than 73 eyes and the differences were statistically significant. (*P* = .003 for horizontal tilt; .038 for vertical tilt by Mann–Whitney *U* test) Figures 1 and 2 show the prediction error according to axial length in femtosecond laser-assisted group and conventional group. The results showed that the axial length was not the cause of the superiority of the femto-assisted group, and the prediction error was not affected by axial length.

To our knowledge, this is the 1st study evaluating the refractive predictability after using the Catalys Precision Laser System. Catalys Precision laser system provides a new method for capsulotomy centration. This laser includes 3-dimensional OCT (3D-OCT) and measures axial and sagittal sectional scanned images. The scanned capsule is an imaginary line of the crystalline lens seen from the anterior and posterior capsule and the midpoint of scanned capsule is used for capsulotomy centration. In the former study^23^, we evaluated OCT, MRI, and anterior segment photographs to investigate which anatomical structure most closely matches the preoperative lens center and provides perfect concordance with the postoperative IOL center and concluded that the scanned capsule center tended to be closer to the IOL center than the pupillary center and limbal center. Schultz et al^[14]^ investigated the overlap between the anterior capsule and IOL optic at the end of cataract surgery and concluded that the percentage of 360 degree overlap was higher in the capsulotomy aligned on the scanned capsule center than the capsulotomy centered on pupil. In this study, we performed femtosecond laser-assisted capsulotomy via scanned capsule center in all cases. It may be assumed that this resulted in better results than manual cataract surgery.

We compared the predictability calculated by the 5 kinds of formulas. Cataract surgeons have aimed to create a formula for determining appropriate IOL power. Popular formulas for IOL power calculation such as SRK/T and Hoffer Q^[15]^ formulas are based on thin lens optics, in which the cornea and the lens (crystalline or IOL) are replaced by infinitely thin lenses with 2 refractive powers. They were designed to generate a more accurate prediction of the effective lens position (ELP) by incorporating the effect of corneal curvature.^[16]^ Sheard et al^[17]^ found a systematic error of the SRK/T formula in its prediction of corneal height and proposed the T2 formula that corrected the prediction process of corneal height with a regression formula derived from a large collection of patient data. The Haigis formula considers preoperative anterior chamber depth instead of preoperative corneal power for the prediction of effective lens position.^[18]^ The Barret-Universal II formula considers both corneal power and anterior chamber depth for ELP prediction. In this study, we did not consider the optional elements including lens thickness and corneal diameter for IOL power calculation. The main purpose of this study was not to analyze which IOL calculation formulas showed high accuracy, so statistical comparisons between formulas were not performed.

Hoffer et al^[19]^ have proposed protocols for studies of IOL formula accuracy and we controlled several factors based on this protocols. Mean errors derived from 2 groups were made to equal 0 in each formula. We also analyzed both mean absolute error and median absolute error. Only one eye of each patient was included and one kind of intraocular lens was analyzed. Postoperative subjective refraction was measured at 3 months after cataract surgery.

This study has some limitations. We evaluated a 1-piece IOL in this study. Savini et al^[20]^ concluded that 3-piece IOLs produced better refractive outcomes than 1-piece IOLs, assuming that the rigid haptics of 3-piece IOLs exert more pressure against the capsular bag than the haptics of 1-piece IOLs. Plate-haptic IOL also showed a higher percentage of 360 degree overlap between anterior capsule and the optic of IOL comparing with 3-piece IOL.^[14]^ Further studies investigating the refractive outcomes after the implantation of 3-piece IOL or plate-haptic IOL are needed as well. Secondly, we did not evaluate the refractive outcomes produced by other centration modes provided by Catalys laser system. In this laser system, the cataract surgeons can select the limbal center and the pupillary center in addition to the scanned capsule center performed in this study.

In conclusion, femtosecond laser-assisted cataract surgery with Catalys femtosecond laser system produced better refractive outcomes than conventional cataract surgery. This technology may yield promising results for cataract surgery.

## Author contributions

**Conceptualization:** Woong-Joo Whang, Young-Sik Yoo, Geunyoung Yoon.

**Data curation:** Woong-Joo Whang.

**Formal analysis:** Woong-Joo Whang.

**Investigation:** Woong-Joo Whang.

**Methodology:** Woong-Joo Whang.

**Project administration:** Choun-Ki Joo.

**Supervision:** Choun-Ki Joo, Geunyoung Yoon.

**Validation:** Young-Sik Yoo.

**Writing – original draft:** Woong-Joo Whang.

**Writing – review & editing:** Young-Sik Yoo.
